# Genome sequences of *Pasteurellaceae* involved in bovine respiratory disease

**DOI:** 10.1128/mra.00352-25

**Published:** 2025-07-31

**Authors:** Daniel Kos, Joseph A. Ross, Nick Allan, Mike Jelinski, Stuart Thiessen, Murray Jelinski, Antonio Ruzzini

**Affiliations:** 1 Department of Large Animal Clinical Sciences, Western College of Veterinary Medicine, University of Saskatchewan 7235, Saskatoon, Saskatchewan, Canada; 2 Chinook Contract Research 607878 , Airdrie, Alberta, Canada; 3 Veterinary Agri-Health Services Ltd, Rock View Country, Alberta, Canada; 4 Namaka Farms, Inc., Strathmore, Alberta, Canada; 5 Department of Veterinary Microbiology, Western College of Veterinary Medicine, University of Saskatchewan 70399, Saskatoon, Saskatchewan, Canada; 6 Department of Biochemistry, Microbiology and Immunology, College of Medicine, University of Saskatchewan 12371, Saskatoon, Saskatchewan, Canada; University of Maryland School of Medicine, Baltimore, Maryland, USA

**Keywords:** bovine resipiratory disease, feedlot cattle, *Histophilus somni*, *Mannheimia haemolytica*, *Pasteurella multocida*, bovine pathogen

## Abstract

We present closed circular genome sequences of *Histophilus somni*, *Mannheimia haemolytica,* and *Pasteurella multocida* associated with bovine respiratory disease in feedlot cattle. The data will contribute to better understanding of disease and the development of potential mitigation strategies.

## ANNOUNCEMENT

Bovine respiratory disease (BRD) is the primary cause of animal treatment and loss at beef feedlots (1–3). Opportunistic bacterial pathogens, including three *Pasteurellaceae* family members, *Histophilus somni, Mannheimia haemolytica,* and *Pasteurella multocida,* commonly contribute to BRD, along with viral co-infection and social and environmental stressors (4).

Seven distinct bacteria – *H. somni*, three *P. multocida,* and three *M. haemolytica –* were isolated from cattle suffering from or lost due to BRD at a feedlot in Alberta, Canada, in November 2020. They originated from six febrile animals, living in four different pens (A–D; Table 1), sampled by deep nasopharyngeal swab (DNS), or necropsy (University of Saskatchewan, Animal Use Protocol 20170021). DNSs were used directly in streak plating on blood agar (tryptic soy agar;TSB, with 5% sheep’s blood) supplemented with 15 μg/mL bacitracin (Dalynn Biological, Calgary, AB, Canada; PB07). A post-mortem lung sample (~2 cm^2^), obtained from the interface between consolidated and unconsolidated area of a lesion, was stomached in liquid media prior to plating on blood agar. Cultivation for isolation was carried out at 37°C in a 5% CO_2_ environment.

**TABLE 1:** Genome sequencing and assembly statistics of the genomes^*a*^

ID [origin], Genbank, SRR no.	ST	Pen	Size (Mbp)	reads	read N50	Coverage	GC (%)	Gene count: coding/RNA/pseudo/ARG
*H. somni*								
CCR1 [DNS], *CP186878.1,* *SRR32971702*	1	A	2.27	74,783	12,285	385	38	2,020/70/42/9
*M. haemolytica*								
CCR2 [DNS], *CP186707.1,* *SRR32971701*	1	A	2.71	59,034	12,207	252	41	2,613/89/86/1
CCR3 [DNS], *CP187427.1,* *SRR32971700*	1	B	2.70	79,586	11,853	336	41	2,602/89/90/1
CCR4 [DNS] *CP187428.1,* *SRR32971699*	1	B	2.70	68,820	10,850	233	41	2,609/89/87/1
*P. multocida*								
CCR5 [DNS] *CP189725.1,* *SRR32971698*	1	B	2.45	93,808	11,719	431	40	2,192/80/32/7
CCR6 [Lung], *CP187520.1,* *SRR32971697*	7	C	2.51	70,773	11,385	306	40	2,346/78/29/1
CCR7 [DNS] *CP187521.1,* *SRR32971696*	7	D	2.51	49,410	12,760	241	40	2,345/79/28/1

^*a*^ ST, sequence type.

To prepare DNA for sequencing, *M. haemolytica* and *P. multocida* were cultured aerobically overnight at 37°C in TSB whereas *H. somni* was cultivated in TSB supplemented with 5% horse serum (v/v) in an oxygen-deprived environment (a sealed culture tube). Cells were harvested by centrifugation at 4,000 *g* and DNA was isolated using the Purelink Microbiome DNA purification kit (Waltham, MA, USA). DNA (1 μg) was sheared by a Megaruptor (Diagenode, NJ, USA) and size selected using a Femto Pulse system (Agilent, CA, USA). Sequencing libraries were constructed and barcoded using the SMRTbell Prep Kit 3.0 and SMRTbell Barcoded Adapter Plate 3.0 before size selection using a Sage Science BluePippin system (Beverly, MA, USA). A PacBio Sequel IIe system and SMRT Cell 8M was employed to generate HiFi reads. Assemblies were performed using Flye (5) for pacbiohifi data and default parameters (v.2.9.5-b1801; Table 1), resulting in circularized, non-rotated genomes (a single chromosome) for each isolate with one exception. Manual curation to generate the final circular, non-rotated CCR5 assembly was required: a 48.7 kb phage-encoding contig was found to have homologous regions (19.8 and 16.8 kb) to the termini of a second 2.4 Mb contig, and the two were merged accordingly. Genes were defined by the NCBI prokaryotic genome annotation pipeline (6), antimicrobial resistance genes (ARGs) were identified (>80% identity match; Table 1) using the Comprehensive Antibiotic Resistance Database (v.4.0.1) through the Resistance Gene Identifier (RGI v.6.0.5) web portal tool (7) and sequence typing (Table 1) was performed using 7-gene PubMLST schemes (8).

Notably, *H. somni* CCR1 encoded for nine ARGs, including *floR*, *msrE*, *mphE,* and *tet(H*), which are known to be involved in resistance to commonly used metaphylactic antibiotics.

## Data Availability

Genome assemblies have been deposited in the NCBI (PRJNA1246242; CP186878.1, CP186707.1, CP187427.1, CP187428.1, CP189725.1, CP187520.1, CPI87521.1). Raw reads were deposited in the Sequence Read Archive (SRP576414; SRR32971702, SRR32971701 SRR32971700, SRR32971699, SRR32971698, SRR32971697, SRR32971696).
